# Regge Models of Proton Diffractive Dissociation Based on Factorisation and Structure Functions

**DOI:** 10.3390/e24071001

**Published:** 2022-07-19

**Authors:** László Jenkovszky, Rainer Schicker, István Szanyi

**Affiliations:** 1 Bogolyubov Institute for Theoretical Physics (BITP), Ukrainian National Academy of Sciences, 14-b, Metrologicheskaya Str., 03680 Kiev, Ukraine; 2 Physikalisches Institut, University Heidelberg, Im Neuenheimer Feld 226, 69120 Heidelberg, Germany; schicker@physi.uni-heidelberg.de; 3 Department of Atomic Physics, Eötvös University, Pázmány P. s. 1/A, 1117 Budapest, Hungary; szanyi.istvan@wigner.hu; 4 Wigner Research Centre for Physics, P.O. Box 49, 1525 Budapest, Hungary; 5 MATE Institute of Technology, Károly Róbert Campus, Mátrai út 36, 3200 Gyöngyös, Hungary

**Keywords:** proton diffractive dissociation, Regge factorisation, structure functions

## Abstract

Recent results by the authors on proton diffractive dissociation (single, double and central) in the low-mass resonance region with emphasis on the LHC kinematics are reviewed and updated. Based on the previous ideas that the contribution of the inelastic proton–Pomeron vertex can be described by the proton structure function, the contribution of the inelastic Pomeron–Pomeron vertex appearing in central diffraction is now described by a Pomeron structure function.

## 1. Introduction

Proton and deuteron diffractive dissociation was intensively studied in the past century at FNAL and CERN-ISR. The relevant experimental results and their phenomenological interpretation were covered in a large number of papers, see Refs. [1,2,3] and references therein. Recent LHC-related developments are discussed, e.g., in Refs. [4,5,6]. The basic idea behind these and similar studies is the identification of the exchanged Pomeron with a flux emitted by the diffractively scattered proton [7].

A different point of view was taken in Refs. [8,9,10,11], where, following C.A. Jaroszkiewicz and P.V. Landshoff [12], the unknown inelastic proton–Pomeron (pP) vertex was associated with the deep inelastic scattering (DIS) photon–nucleon structure function (SF), known from the experiments at HERA. In doing so, G.A. Jaroszkiewicz and P.V. Landshoff [12] used a high-energy, Regge-behaved formula for the DIS SF, leaving outside the low-energy (missing mass) resonance structure. Resonances were included in this formalism in a series of papers [8,9,10,11], where, by duality, the high-energy behaviour of the SF was replaced by its low-energy (missing mass) SF, dominated by direct channel non-linear complex Regge trajectories, producing finite-width resonances. Now we extend the structure function formalism to the inelastic Pomeron–Pomeron (PP) vertex to model central diffractive processes.

Diffractive dissociation is interesting and important for many reasons. One is that new experimental data are expected from the ongoing LHC run, especially in the central region, which will help us fix the remaining freedom/flexibility of the models. On the other hand, the predictions of the model may guide experimentalists in tuning their detectors. Furthermore, it is important to remember that the high energies, typical of the LHC make it possible to neglect—in most of the kinematical configurations—the contribution from secondary reggeons and allow us to use Regge factorisation and concentrate on the nature of the Pomeron.

The paper is organised as follows: in Section 2 models of differential cross-sections of the diffractive processes, including elastic scattering as well as single, double and central diffractive dissociation are constructed. In Section 3, the treatment of the pP and PP vertices is introduced based on the formalism of structure functions. The calculated integrated cross-sections for processes with diffractive dissociation, including fits to the available measured data, are presented in Section 4. The calculated differential cross-sections are presented in Section 5. Our results and the conclusions are summarised in Section 6.

## 2. Differential Cross-Sections

In this section, we summarise and update the basic formulae for elastic scattering, single diffractive dissociation and double diffractive dissociation (elaborated in a series of papers [8,9,10,11,13]), we also extend the formalism based on the use of structure functions to central diffractive dissociation and mixed processes. This is an important step on the way towards the elaboration of a unique and adequate language and relevant set of variables and measurables, understandable and convenient both for theorists and experimentalists.

**Figure 1:**
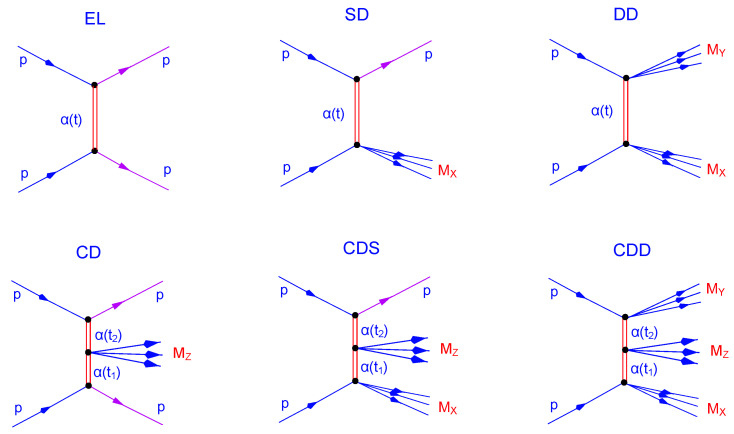
Diffraction: elastic scattering (EL); single (SD), double (DD) and central (CD) dissociation; mixed central and single dissociation (CDS); mixed central and double dissociation (CDD).

Figure 1 shows the main topologies appearing in diffractive dissociation under discussion. It may serve also as a guide to relevant equations that follow.

The differential cross-section of elastic proton–proton scattering (*EL*) is:

(1) $$\frac{d\sigma_{EL}}{dt}=A_{EL}\beta^{2}\left(t\right)\beta^{2}\left(t\right){\left|\eta \left(t\right)\right|}^{2}{\left(\frac{s}{s_{0}}\right)}^{2\alpha_{P}\left(t\right)-2},$$

where *s* and *t* are the Mandelstam variables. $A_{i}$ with i=EL and, later in the text, i∈{SD,DD,CD,CDS,CDD} are free parameters of dimension $\left[A_{i}\right]$ = mb/GeV^2^, including also normalisation constants. The proton–Pomeron coupling squared is: $\beta^{2}\left(t\right)=e^{bt}$, where *b* is a free parameter and b≈1.97 GeV^2^ determined in Ref. [13]. The Pomeron trajectory is $\alpha_{P}\left(t\right)=1+\epsilon +\alpha^{\prime }t$, where ϵ≈0.08 and $\alpha^{\prime }\approx 0.3$ GeV^2^ [13]. The signature factor is $\eta \left(t\right)=e^{-i\frac{\pi }{2}\alpha_{P}\left(t\right)}$; its contribution to the differential cross-section is ${\left|\eta \left(t\right)\right|}^{2}=1$, therefore we ignore it in what follows. We set $s_{0}=1$ GeV^2^ for simplicity.

The differential cross-section of proton–proton single diffraction (SD) is:

(2) $$2\cdot \frac{d^{2}\sigma_{SD}}{dtdM_{X}^{2}}=A_{SD}\beta^{2}\left(t\right){\tilde{W}}_{2}^{Pp}\left(M_{X}^{2},t\right){\left(\frac{s}{M_{X}^{2}}\right)}^{2\alpha_{P}\left(t\right)-2}\;,$$

where ${\tilde{W}}_{2}^{Pp}\left(M_{X}^{2},t\right)$ is related to the proton SF, $F_{2}^{p}\left(M_{X}^{2},t\right)$ (see Section 3 for details).

From Figure 1, the differential cross-section of proton–proton double diffraction (DD) is:

(3) $$\frac{d^{3}\sigma_{DD}}{dtdM_{X}^{2}dM_{Y}^{2}}=A_{DD}{\tilde{W}}_{2}^{Pp}\left(M_{X}^{2},t\right){\tilde{W}}_{2}^{Pp}\left(M_{Y}^{2},t\right){\left(\frac{ss_{0}}{M_{X}^{2}M_{Y}^{2}}\right)}^{2\alpha_{P}\left(t\right)-2},$$

where ${\tilde{W}}_{2}^{Pp}\left(M_{X}^{2},t\right)$ is the same function as that used in the SD reaction, with corresponding arguments.

Accordingly, the differential cross-sections of proton–proton central diffraction (CD), central diffraction with single diffraction (CDS) and central diffraction with double diffraction (CDD) are:

(4) $$\frac{d^{4}\sigma_{CD}}{dt_{1}dt_{2}d\xi_{1}d\xi_{2}}=A_{CD}\beta^{2}\left(t_{1}\right)\beta^{2}\left(t_{2}\right){\tilde{W}}_{2}^{PP}\left(M_{Z}^{2},t_{1},t_{2}\right)\xi_{1}^{1-2\alpha_{P}\left(t_{1}\right)}\xi_{2}^{1-2\alpha_{P}\left(t_{2}\right)}\;,$$

(5) $$\begin{array}{c} 2\cdot \frac{d^{5}\sigma_{CDS}}{dt_{1}dt_{2}d\xi_{2}d\xi_{2}dM_{X}^{2}}=A_{CDS}\beta^{2}\left(t_{2}\right){\tilde{W}}_{2}^{Pp}\left(M_{X}^{2},t_{1}\right){\tilde{W}}_{2}^{PP}\left(M_{Z}^{2},t_{1},t_{2}\right)\\ \times \xi_{1}^{1-2\alpha_{P}\left(t_{1}\right)}{\left(\frac{s_{0}}{M_{X}^{2}}\right)}^{2\alpha_{P}\left(t_{1}\right)+2}\xi_{2}^{1-2\alpha_{P}\left(t_{2}\right)}\;, \end{array}$$

(6) $$\begin{array}{c} \frac{d^{6}\sigma_{CDD}}{dt_{1}dt_{2}d\xi_{2}d\xi_{2}dM_{X}^{2}dM_{Y}^{2}}=A_{CDD}{\tilde{W}}_{2}^{Pp}\left(M_{X}^{2},t_{1}\right){\tilde{W}}_{2}^{Pp}\left(M_{Y}^{2},t_{2}\right){\tilde{W}}_{2}^{PP}\left(M_{Z}^{2},t_{1},t_{2}\right)\\ \times \xi_{1}^{1-2\alpha_{P}\left(t_{1}\right)}{\left(\frac{s_{0}}{M_{X}^{2}}\right)}^{2\alpha_{P}\left(t_{1}\right)+2}\xi_{2}^{1-2\alpha_{P}\left(t_{2}\right)}{\left(\frac{s_{0}}{M_{Y}^{2}}\right)}^{2\alpha_{P}\left(t_{2}\right)+2}\;, \end{array}$$

where ${\tilde{W}}_{2}^{PP}\left(M_{X}^{2},t\right)$ is the contribution of the inelastic PP vertex to the differential cross-section related to the Pomeron SF, $F_{2}^{P}\left(M_{Z}^{2},t\right)$ as explained in Section 3.

If there are two incoming protons with four-momenta $p_{1}$ and $p_{2}$, then $\xi_{1}p_{1}$ four-momentum is carried by one of the two Pomerons and $\xi_{2}p_{2}$ four-momentum is carried by the other one. Consequently, the squared mass of the centrally produced system is: $M_{Z}^{2}={\left(\xi_{1}p_{1}+\xi_{2}p_{2}\right)}^{2}=\left(\xi_{1}^{2}+\xi_{2}^{2}\right)m_{p}^{2}$$+2\xi_{1}\xi_{2}\left(s/2-m_{p}^{2}\right)$, where $m_{p}$ is the mass of the proton. Using the fact that $m_{p}^{2}\ll s$, one has: $M_{Z}^{2}\approx \xi_{1}\xi_{2}s$.

Note also that $t_{1}$ and $t_{2}$ are connected to the virtualities of the colliding Pomerons: $Q_{1}^{2}=-q_{1}^{2}=-t_{1}$ and $Q_{2}^{2}=-q_{2}^{2}=-t_{2}$, where $Q_{1}$ and $Q_{2}$ are the virtualities and $q_{1}$ and $q_{2}$ are the four momenta of the Pomerons.

## 3. The Inelastic *Pp* and *PP* Vertices

Following Refs. [8,9,10], we write the Pomeron–proton vertices as:

(7) $${\tilde{W_{2}}}^{Pp}\left(M_{X}^{2},t\right)\equiv \frac{W_{2}^{Pp}\left(M_{X}^{2},t\right)}{2m_{p}},$$

where:

(8) $$W_{2}^{Pp}\left(M_{X}^{2},t\right)=\frac{F_{2}^{p}\left(M_{X}^{2},t\right)}{\nu (M_{X}^{2},t)},\;\;\;\;\;\;\;\;\;F_{2}^{p}\left(M_{X}^{2},t\right)=\frac{-t(1-x)}{4\pi \alpha (1-4m_{p}^{2}x^{2}/t)}\sigma_{t}^{Pp}\left(M_{X}^{2},t\right)\;,$$

$\sigma_{t}^{Pp}$ is the total Pomeron–proton cross-section, $m_{p}$ is the mass of the proton, α is the fine structure constant,

(9) $$x\equiv x\left(M_{X}^{2},t\right)=\frac{-t}{M_{X}^{2}-t-m_{p}^{2}},$$

and

(10) $$\nu \left(M_{X}^{2},t\right)=\frac{-t}{2m_{p}x\left(M_{X}^{2},t\right)}.$$

The total Pp cross-section is:

(11) $$\sigma_{t}^{Pp}\left(M_{X}^{2},t\right)=\sigma_{t,0}^{Pp}\left(M_{X}^{2}\right)+\sigma_{t,\mathrm{res}}^{Pp}\left(M_{X}^{2},t\right),$$

where:

(12) $$\sigma_{t,0}^{Pp}\left(M_{X}^{2}\right)=\sigma_{0}\tau^{8}\left(M_{X}^{2}\right){\left(\frac{M_{X}^{2}}{s_{0}}\right)}^{\epsilon },$$

and, according to the optical theorem,

(13) $$\sigma_{t,\mathrm{res}}^{Pp}\left(M_{X}^{2},t\right)=\frac{8\pi }{P_{CM}M_{X}}\;ℑm\;A_{\mathrm{res}}^{Pp}\left(M_{X}^{2},\tilde{t}=0\right)\;,$$

with $\sigma_{0}=2.82\;mb$ or $7.249\;{GeV}^{-2}$ [6],

$$\tau \left(M_{X}^{2}\right)=\frac{e^{-M_{X}^{2}/m_{0}^{2}}-1}{e^{-M_{X}^{2}/m_{0}^{2}}+1},\;\;\;\;\;\;m_{0}^{2}=1\;{\mathrm{GeV}}^{2},$$

$$P_{CM}\equiv P_{CM}\left(M_{X}^{2},t\right)=\frac{M_{X}^{2}-m_{p}^{2}}{2(1-x)}\sqrt{\frac{1-4m_{p}^{2}x^{2}/t}{M_{X}^{2}}},$$

where *x* is defined by Equation (9). In $\sigma_{t,0}^{Pp}\left(M_{X}^{2}\right)$, $\tau (M_{X}^{2})$ to the power of 8 is included. This provides a sharp enough suppression for $\sigma_{t,0}^{Pp}\left(M_{X}^{2}\right)$ in the kinematical region where no dissociation occurs, $M_{X}^{2}<{\left(m_{p}+m_{\pi^{0}}\right)}^{2}$, and also in the low $M_{X}^{2}$ region where dissociation occurs but resonances do not appear.

Note that $t\neq \tilde{t}$. *t* is connected to the virtuality of the radiated particle, the Pomeron, in the pp→Xp process, $Q^{2}=-q^{2}=-t$, where *q* is the four-momentum of the Pomeron. $\tilde{t}$ is the squared four-momentum transfer in the Pp→Pp process. Hence, by the optical theorem, $\sigma_{t,res}^{Pp}=ℑm\;A_{\mathrm{res}}^{Pp}\left(M_{X}^{2},\tilde{t}=0\right)$ up to normalisation, where $ℑm\;A_{\mathrm{res}}^{Pp}$ is the imaginary part of the Pp scattering amplitude that includes the resonances. According to Refs. [8,9], the latter is given as:

(14) $$ℑm\;A_{\mathrm{res}}^{Pp}\left(M_{X}^{2},\tilde{t}\right)=\sum_{J}\frac{{\left[f\left(\tilde{t}\right)\right]}^{J+3/2}ℑm\alpha_{N^{*}}\left(M_{x}^{2}\right)}{{\left(J-\;ℜe\alpha_{N^{*}}\left(M_{x}^{2}\right)\right)}^{2}+{\left(ℑm\;\alpha_{N^{*}}\left(M_{x}^{2}\right)\right)}^{2}},$$

where $\alpha_{N^{*}}$ is the nucleon trajectory,

(15) $$f\left(\tilde{t}\right)={\left(1-\tilde{t}/t_{0}\right)}^{-2},$$

and $t_{0}=0.71$ GeV^2^.

The explicit form of the nucleon trajectory is given in Refs. [8,10]. Resonances on this trajectory appear with total spins J=5/2,9/2,13/2, …

The contribution from the PP vertex to the differential cross-section is:

(16) $${\tilde{W_{2}}}^{PP}\left(M_{Z}^{2},t_{1},t_{2}\right)\equiv \frac{F_{2}^{P}\left(M_{Z}^{2},t_{1},t_{2}\right)}{\nu^{P}\left(M_{Z}^{2},t_{1},t_{2}\right)},$$

where:

(17) $$F_{2}^{P}\left(M_{Z}^{2},t_{1},t_{2}\right)=\frac{\nu^{P}\left|t_{1}\right|}{4\pi^{2}\alpha \sqrt{{\left(\nu^{P}\right)}^{2}-t_{1}t_{2}}}\sigma_{t}^{PP}\left(M_{Z}^{2},t_{1},t_{2}\right)\;,$$

is the Pomeron structure function based on the structure function of the virtual photon given in Ref. [14], and

(18) $$\nu^{P}\equiv \nu^{P}\left(M_{Z}^{2},t_{1},t_{2}\right)=\frac{1}{2}(M_{Z}^{2}-t_{1}-t_{2}).$$

The total PP cross-section is:

(19) $$\sigma_{t}^{PP}\left(M_{Z}^{2},t_{1},t_{2}\right)=\sigma_{t,0}^{PP}\left(M_{Z}^{2}\right)+\sigma_{t,\mathrm{res}}^{PP}\left(M_{Z}^{2},t_{1},t_{2}\right),$$

where $\sigma_{t,0}^{PP}$ is identified by $\sigma_{t,0}^{Pp}$ as in Ref. [6],

(20) $$\sigma_{t,\mathrm{res}}^{PP}\left(M_{Z}^{2},t_{1},t_{2}\right)=\frac{8\pi }{P_{CM}\sqrt{M_{Z}^{2}}}\;Im\;A_{\mathrm{res}}^{PP}\left(M_{Z}^{2},\tilde{t}=0\right)\;,$$

$P_{CM}\equiv P_{CM}\left(M_{Z}^{2},t_{1},t_{2}\right)=\frac{M_{Z}^{2}-t_{1}}{2\left(1+\frac{t_{2}}{2\nu^{P}}\right)}\sqrt{\frac{1+t_{1}t_{2}/{\left(\nu^{P}\right)}^{2}}{M_{Z}^{2}}}$ and $\nu^{P}$ is given by Equation (18). Based on Ref. [15]:

(21) $$ℑm\;A_{\mathrm{res}}^{PP}\left(M_{Z}^{2},\tilde{t}\right)=\sum_{i=f,P}\sum_{J}\frac{{\left[f_{i}\left(\tilde{t}\right)\right]}^{J+2}ℑm\;\alpha_{i}\left(M_{Z}^{2}\right)}{{\left(J-ℜe\;\alpha_{i}\left(M_{Z}^{2}\right)\right)}^{2}+{\left(ℑm\;\alpha_{i}\left(M_{Z}^{2}\right)\right)}^{2}},$$

where the index *i* runs over the trajectories, which contributes to the amplitude. For all trajectories, we sum over the states with full spins *J*. The $f_{i}\left(\tilde{t}\right)$ is the pole residue and given by Equation (15) for all trajectories uniformly. Note that $\tilde{t}$ is the squared four-momentum transfer in the PP→PP process while $t_{1}$ and $t_{2}$ are connected to the virtualities of the colliding Pomerons.

The $PP\to M_{Z}^{2}$ Pomeron–Pomeron channel couples to the Pomeron and the *f*-meson by the conservation of the quantum numbers. The explicit form of the Pomeron trajectory can be found in Ref. [13], while that of the *f*-meson trajectories are given in Ref. [15]. At the present stage of research we include only glueballs lying on the Pomeron trajectory. Ordinary mesons will be added in a forthcoming study.

## 4. Integrated Cross-Sections

In this section, integrated cross-sections for the SD, DD and CD reactions are presented. Numerical calculations for CDS and CDD processes are postponed to a later study.

**Figure 2:**
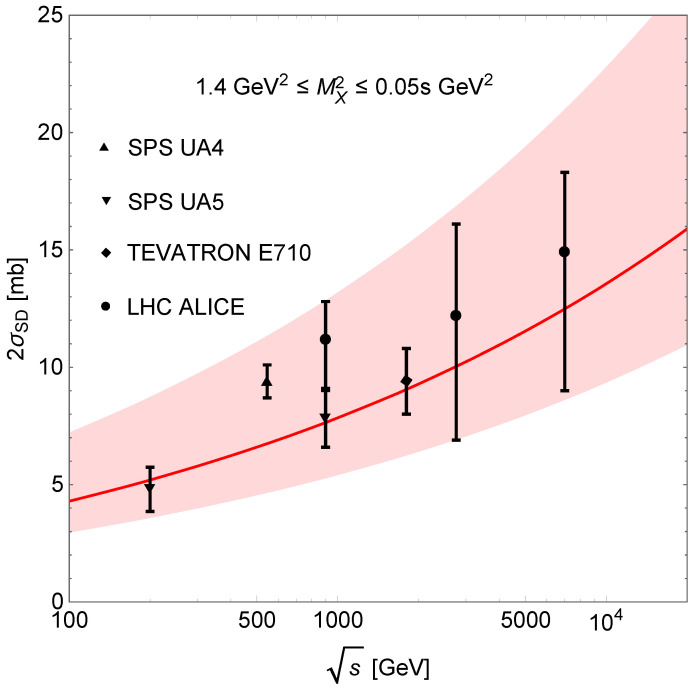
Integrated SD cross-section. The shaded area corresponds to the uncertainty arising from the normalisation parameter $A_{SD}$.

For SD we have:

(22) $$2\sigma_{SD}=\int_{{M_{X}^{2}}_{min}}^{{M_{X}^{2}}_{max}}dM_{X}^{2}\int_{t_{min}}^{t_{max}}dt\;2\cdot \frac{d\sigma_{SD}^{2}}{dM_{X}^{2}dt},$$

where ${M_{X}^{2}}_{min}=1.4$ GeV^2^ [4], ${M_{X}^{2}}_{max}=0.05s$ GeV^2^, $t_{min}=-\infty$ and $t_{max}=0$ GeV^2^ (practically $t_{min}=-1$ GeV^2^). The result is shown in Figure 2 with $A_{SD}=0.063_{-0.020}^{+0.043}$ mb/GeV^2^ resulting from a fit to the experimental data. The theoretical uncertainties in Figure 2 are correlated with the errors in the data.

**Figure 3:**
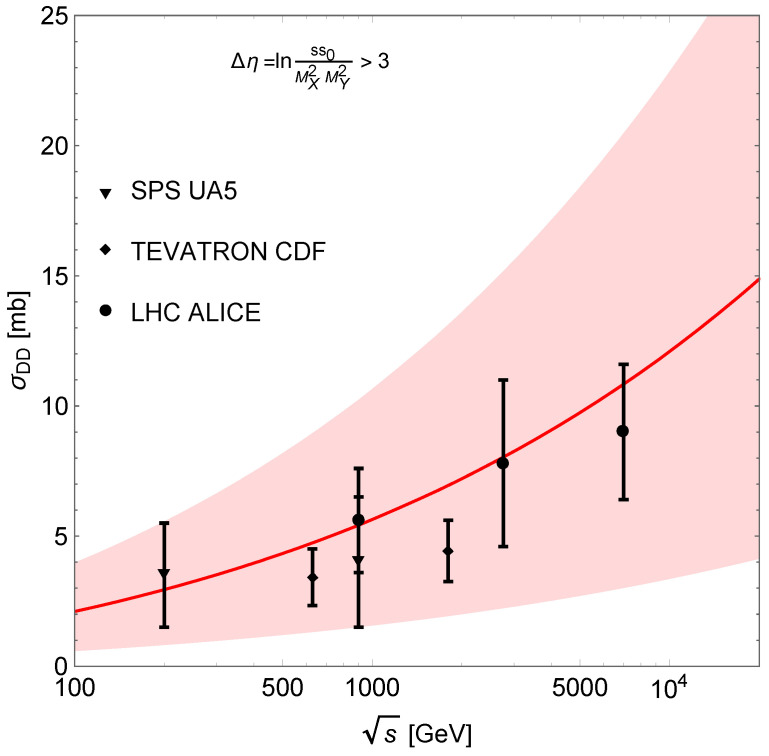
Integrated DD cross-section. The shaded area corresponds to the uncertainty arising from the normalisation parameter $A_{DD}$.

For DD one has [4]:

(23) $$\sigma_{DD}=\int_{{M_{X}^{2}}_{min}}^{{M_{X}^{2}}_{max}}dM_{X}^{2}\int_{{M_{Y}^{2}}_{min}}^{{M_{Y}^{2}}_{max}}dM_{Y}^{2}\int_{t_{min}}^{t_{max}}dt\frac{d\sigma_{DD}^{3}}{dM_{X}^{2}dM_{Y}^{2}dt},$$

where ${M_{X}^{2}}_{min}=1.4$ GeV^2^, ${M_{X}^{2}}_{max}=0.05ss_{0}/{M_{Y}^{2}}_{min}$ GeV^2^, ${M_{Y}^{2}}_{min}=1.4$ GeV^2^, ${M_{Y}^{2}}_{max}=0.05ss_{0}/{M_{Y}}_{min}^{2}$ GeV^2^, $s_{0}=1$ GeV^2^, $t_{max}=0$ GeV^2^ and $t_{min}=-\infty$. The result is shown in Figure 3 with $A_{DD}=9_{-6.5}^{+8.0}\times 10^{-5}$ mb/GeV^2^ resulting from a fit to the experimental data.

For CD it is convenient to use the variables $\Delta \eta =\ln\frac{s}{M_{Z}^{2}}$ (pesudorapidity-gap) and $\eta_{c}$ (the center of the centrally-produced system in pesudorapidity, η) [3,4]:

(24) $$\begin{array}{c} \frac{d^{4}\sigma_{CD}}{dt_{1}dt_{2}d\Delta \eta d\eta_{c}}=A_{CD}\beta^{2}\left(t_{1}\right)\beta^{2}\left(t_{2}\right){\tilde{W}}_{2}^{PP}(se^{-\Delta \eta },t_{1},t_{2})\\ \times e^{\frac{1}{2}\left[\alpha_{P},\left(t_{1}\right),-,1\right]\left[\Delta ,\eta ,+,\eta_{c}\right]}e^{\frac{1}{2}\left[\alpha_{P},\left(t_{2}\right),-,1\right]\left[\Delta ,\eta ,-,\eta_{c}\right]}\;. \end{array}$$

Now, the integrated cross-section for CD is:

(25) $$\sigma_{CD}=\int_{{t_{1}}_{min}}^{{t_{1}}_{max}}dt_{1}\int_{{t_{2}}_{min}}^{{t_{2}}_{max}}dt_{2}\int_{\Delta \eta_{min}}^{\Delta \eta_{max}}\int_{{\eta_{c}}_{min}}^{{\eta_{c}}_{max}}\frac{d^{4}\sigma_{CD}}{dt_{1}dt_{2}d\Delta \eta d\eta_{c}},$$

where ${t_{1}}_{min}={t_{2}}_{min}=-\infty$, ${t_{1}}_{max}={t_{2}}_{max}=0$ GeV^2^, $\Delta \eta_{min}=3$, $\Delta \eta_{max}=\ln\left(s/s_{0}\right)$, $s_{0}=1$ GeV^2^, ${\eta_{c}}_{min}=-\frac{1}{2}\left(\Delta \eta -\Delta \eta_{min}\right)$ and ${\eta_{c}}_{max}=\frac{1}{2}\left(\Delta \eta -\Delta \eta_{min}\right)$ [6].

**Figure 4:**
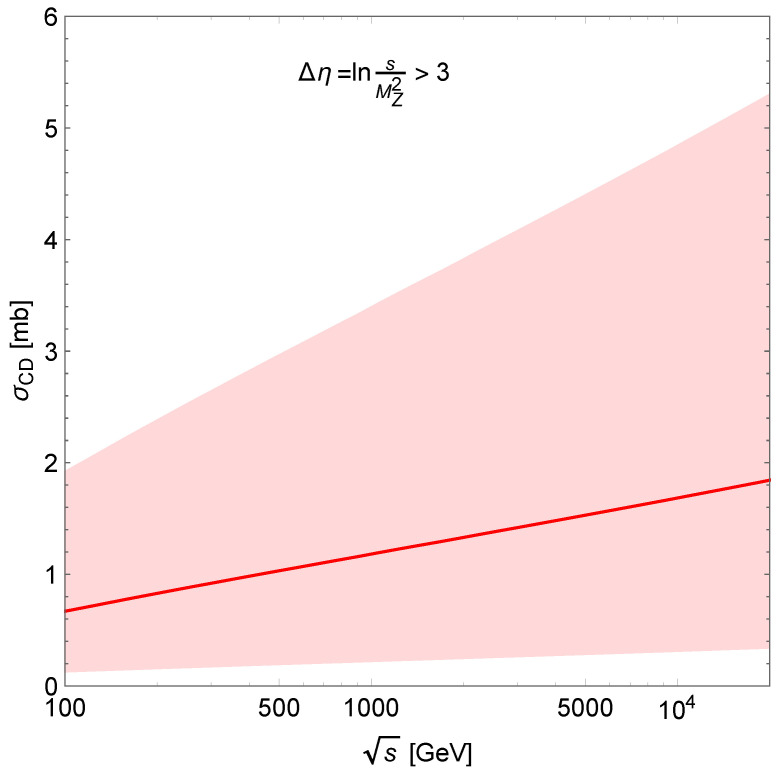
Integrated CD cross-section. The shaded area corresponds to uncertainties inherent in the normalisation parameter $A_{CD}$.

The results are shown in Figure 4 with $A_{CD}=0.066_{-0.54}^{+0.124}$ mb/GeV^2^. The value of this normalisation parameter is obtained using the relation $\sigma_{CD}\approx \frac{{\left(2\sigma_{SD}\right)}^{2}}{\sigma_{tot}^{pp}}$ based on Regge factorisation. The uncertainty is obtained by the calculated uncertainty of $2\sigma_{SD}$ and the total experimental uncertainty of $\sigma_{tot}^{pp}$ [16] at 7 TeV.

## 5. Predictions for Differential Cross-Sections

This section is devoted to our predictions for SD, DD and CD multiple differential cross-sections at $\sqrt{s}=14$ TeV in the low-mass region.

**Figure 5:**
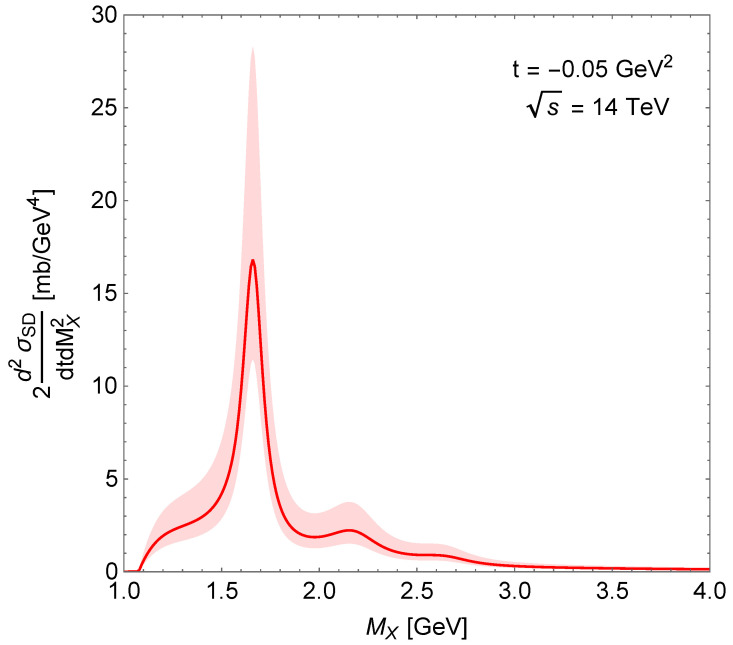
Mass dependence of the SD double differential cross-section.

**Figure 6:**
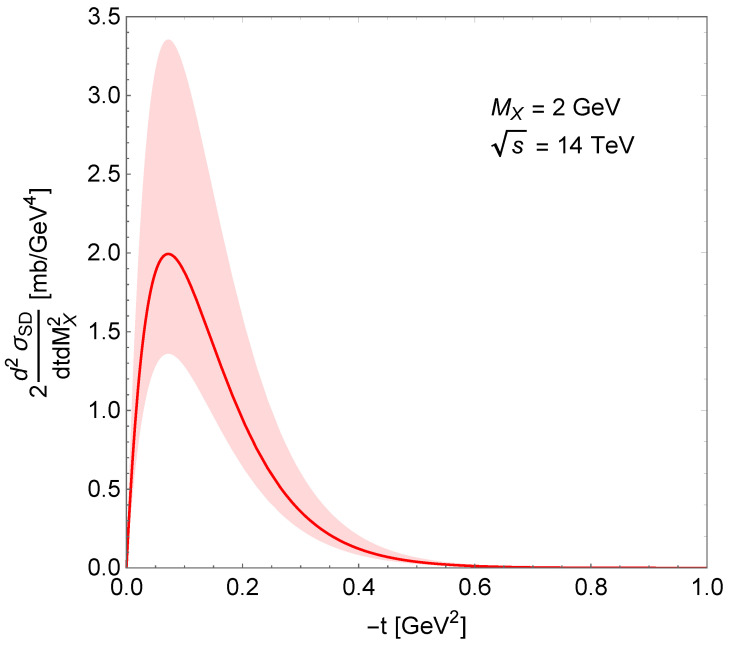
Squared momentum transfer dependence of the SD double differential cross-section.

The $M_{X}$ dependence of SD double differential cross-section is shown in Figure 5. The visible peaks correspond to nucleon resonances: $N^{*}\left(1680\right)$, $N^{*}\left(2220\right)$ and $N^{*}\left(2700\right)$. Figure 6 shows the squared momentum transfer dependence of this cross-section: a peak at low-|t| followed by the usual exponential decrease. The shaded areas around the curves show the uncertainty of the calculations following from the uncertainty of the normalisation parameter.

**Figure 7:**
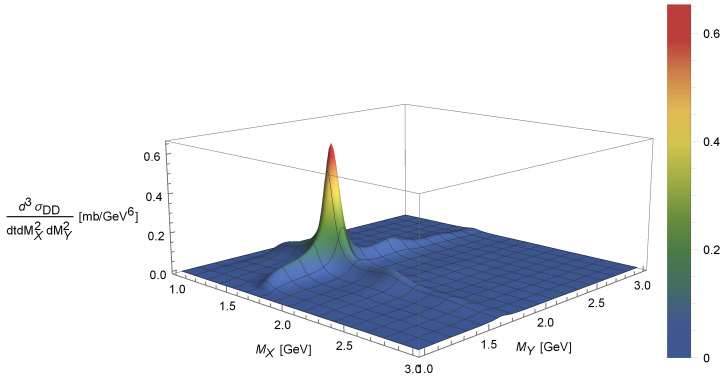
Mass dependence of the DD triple differential cross-section.

**Figure 8:**
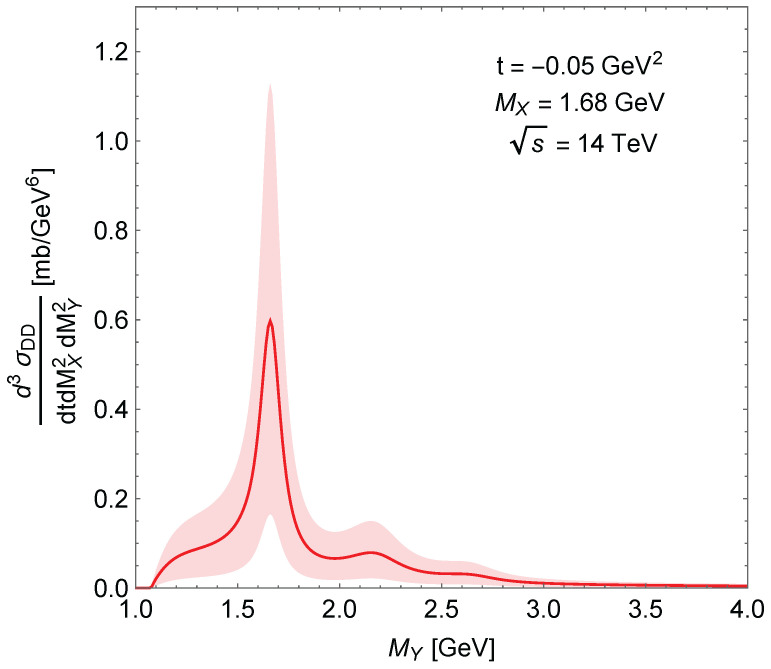
Same as Figure 7 calculated at $M_{X}=1.68$ GeV.

The $M_{X}$ and $M_{Y}$ dependence of the DD triple differential cross-section is shown in Figure 7 as a surface. Similar to SD, the peaks correspond to nucleon resonances: $N^{*}\left(1680\right)$, $N^{*}\left(2220\right)$ and $N^{*}\left(2700\right)$. Figure 8 is a “slice” of Figure 7 corresponding to a fixed $M_{X}$ showing the uncertainty of the calculation originating from the uncertainty of the normalisation parameter.

**Figure 9:**
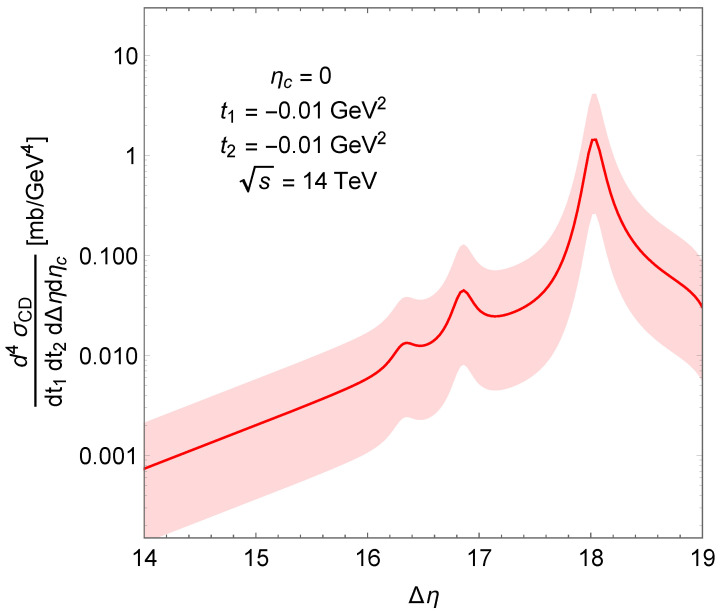
Pseudorapidity-gap dependence of the CD quadruple differential cross-section.

The Δη dependence of the CD quadruple differential cross-section is shown in Figure 9. The visible peaks correspond to glueball resonances lying on the Pomeron trajectory: $J^{PC}=2^{++},\;4^{++}$ and $6^{++}$. Mesons will be included in a forthcomng study.

## 6. Summary

In this paper, we presented updated results on modelling single and double diffraction as well as novel results on modelling central diffraction. The modelling is based on Regge factorisation accompanied by the identification of the contributions of inelastic vertices by structure functions.

We stress that one of the main unknown objects is the inelastic Pp vertex. As mentioned in the Introduction, in most of the papers on the subject, e.g., in Refs. [1,2,3,4,5], one associates (following the ideas of Ref. [7]) the Pomeron with a flux radiated by the incoming proton. The authors of Refs. [8,9,10], following [12], take a different viewpoint and identify the inelastic Pp vertex with the proton SF, known from deep-inelastic electron–proton scattering [17]. In Refs. [8,9,10,11], this SF is specified by the direct-channel resonance diagrams dominated by relevant baryon trajectories producing excited nucleon states (mainly $N^{*}$ resonances).

A completely novel result of this paper is the identification of the inelastic PP vertex with a Pomeron SF. The Pomeron SF is constructed based on the virtual photon SF [14] in a way it can contain mesonic and glueball resonances. The treatment of the inelastic PP vertex is crucial in central diffractive dissociation (diagrams 4–6 in Figure 1). They contain a subdiagram corresponding to collision of two Pomerons (or, more generally, reggeons). Construction of amplitudes describing scatting of virtual hadrons (by “virtual hadrons” we mean states lying on the Pomeron (or any reggeon) trajectory) is of course an open problem. Our present approach is one possibility, although experimental data on central diffraction is needed for justification or for further guide in theoretical developments.

Finally, we highlight that the main part of the dynamics in diffractive dissociation is carried by the Regge trajectories, i.e., nonlinear complex functions. The construction of explicit models of such trajectories is a basic part of this approach, deserving further studies.

## Data Availability

Not applicable.
